# The Value of Whole-Tumor Texture Analysis of ADC in Predicting the Early Recurrence of Locally Advanced Cervical Squamous Cell Cancer Treated With Concurrent Chemoradiotherapy

**DOI:** 10.3389/fonc.2022.852308

**Published:** 2022-05-20

**Authors:** Xiaomiao Zhang, Qi Zhang, Lizhi Xie, Jusheng An, Sicong Wang, Xiaoduo Yu, Xinming Zhao

**Affiliations:** ^1^ Department of Radiology, National Cancer Center/National Clinical Research Center for Cancer/Cancer Hospital, Chinese Academy of Medical Sciences and Peking Union Medical College, Beijing, China; ^2^ GE Healthcare, MR Research, Beijing, China; ^3^ Department of Gynecologic Oncology, National Cancer Center/National Clinical Research Center for Cancer/Cancer Hospital, Chinese Academy of Medical Sciences and Peking Union Medical College, Beijing, China

**Keywords:** cervical squamous cell cancer, FIGO stage, apparent diffusion coefficient, recurrence, concurrent chemoradiotherapy

## Abstract

**Objectives:**

To investigate the value of whole-tumor texture analysis of apparent diffusion coefficient (ADC) map in predicting the early recurrence of patients with locally advanced cervical squamous cell cancer (LACSC) treated with concurrent chemoradiotherapy (CCRT) and establish a combined prediction model including clinical variables and first-order texture features.

**Methods:**

In total, 219 patients (training: n = 153; testing: n = 66) with stage IIB-IVA LACSC treated by CCRT between January 2014 and December 2019 were retrospectively enrolled in this study. Clinical variables and 22 first-order texture features extracted from ADC map were collected. The Mann-Whitney U test or independent sample t test, chi-square test or Fisher’s exact were used to analyze statistically significant parameters, logistic regression analysis was used for multivariate analysis, and receiver operating characteristic analysis was used to compare the diagnostic performance.

**Results:**

In the clinical variables, T stage and lymph node metastasis (LNM) were independent risk factors, and the areas under the curve (AUCs) of the clinical model were 0.697 and 0.667 in the training and testing cohorts, the sensitivity and specificity were 48.8% and 85.5% in the training cohort, and 84.1% and 51.1% in the testing cohort, respectively. In the first-order texture features, mean absolute deviation (MAD) was the independent protective factor, with an AUC of 0.756 in the training cohort and 0.783 in the testing cohort. The sensitivity and specificity were 95.3% and 52.7% in the training cohort and 94.7% and 53.2% in the testing cohort, respectively. The combined model (MAD, T stage, and LNM) was established, it exhibited the highest AUC of 0.804 in the training cohort and 0.821 in the testing cohort, which was significantly higher than the AUC of the clinical prediction model. The sensitivity and specificity were 67.4% and 85.5% in the training cohort and 94.7% and 70.2% in the testing cohort, respectively.

**Conclusions:**

The first-order texture features of the ADC map could be used along with clinical predictive biomarkers to predict early recurrence in patients with LACSC treated by CCRT.

## Introduction

Cervical cancer is the fourth most common cancer affecting women worldwide and is still a major health problem that threatens most women, especially those in developing countries. There were about 604,000 new cases and 342,000 deaths worldwide in 2020 (1). Cervical squamous cell cancer is the most common type of cervical cancer. According to the National Comprehensive Cancer Network (NCCN) Clinical Practice Guidelines in Oncology, concurrent chemoradiotherapy (CCRT) is suggested as the standard treatment strategy for patients with locally advanced cervical carcinoma (LACC) (i.e., those with stage IIB-IVA according to the new 2018 International Federation of Gynecology and Obstetrics [FIGO] staging system) (2, 3). Although multimodality treatment has been reported to improve the outcomes, the overall recurrence rate in patients with LACC is 35%, and the majority of recurrences are detected within 2 years of primary treatment, and the median survival after recurrence is 10-12 months (4–6). For women with recurrent cervical cancer, the patients are likely to develop resistance to first-line chemotherapy drugs, and there are many complications related to surgery and radiotherapy, which is an important cause of death in cervical cancer patients. The prediction of cervical cancer outcomes is one of the most challenging tasks as the management of cervical cancer involves the most complicated cancer treatment strategies. Early and accurate prediction of clinical outcomes would guide clinicians to optimize the treatment and follow-up program in advance, and increase the long-term survival probability.

Conventional magnetic resonance imaging (MRI) is the preferred imaging modality for assessing primary tumor characteristics owing to its excellent soft-tissue contrast. It plays an essential role in the initial staging of disease, but also in therapeutic strategy, in plan treatment, and in evaluating tumor response (7). However, it lacks quantitative parameters reflecting tissue microstructure. Diffusion-weighted imaging (DWI) is a routinely used functional MRI method, which is sensitive to the diffusion of water molecules in tissue and helpful in the tumor diagnosis and characterization by reflecting pathological tumor processes at the microscopic level (8, 9). Additionally, the quantitative assessment can be performed by the measurement of the apparent diffusion coefficient (ADC) (10). The results regarding ADC values in predicting the recurrence of cervical cancer vary greatly (11–15). Some studies showed low percentile ADC values were related to recurrence and a poor survival rate (11), while some found high percentile ADC values significantly associated with recurrence of LACC (12–15). One of the possible reasons might be the choice of region of interest (ROI), as some studies used maximum single slices instead of whole tumor slices.

Texture analysis based on ADC maps is an emerging modality, which can more precisely reflect intratumoral heterogeneity (16). Studies about texture analysis did achieve inspiring results in predicting the clinical outcome of cervical cancer (7, 17, 18), but large differences exist between studies. The use of a large number of radiomic metrics and the lack of uniformity of these measures and their selective use, which may be correlated, have led to studies with results that are nonreproducible and noncomparable (19). Lin et al. found that first-order texture features extraction from whole tumor volume were robust and reproducible and could be used for longitudinal monitoring of treatment response of LACC (20).

Although the FIGO staging system is considered the most powerful prognostic parameter, other clinical variables have been shown to have additional significance in predicting the prognosis of patients with cervical cancer (21, 22). Rose et al. (22) evaluated the prognostic factors for patients with LACC treated with CCRT and found that FIGO stage, tumor maximum diameter, tumor grade, and lymph node status were significantly associated with tumor recurrence.

This study aimed to (a) investigate the value of first-order texture features extracted from ADC maps in predicting the early recurrence of patients with locally advanced cervical squamous cell cancer (LACSC) treated with CCRT, (b) further establish a combined prediction model including clinical variables and first-order texture features, and (c) evaluate its prediction performance.

## Materials and Methods

### Subjects

**Figure 1:**
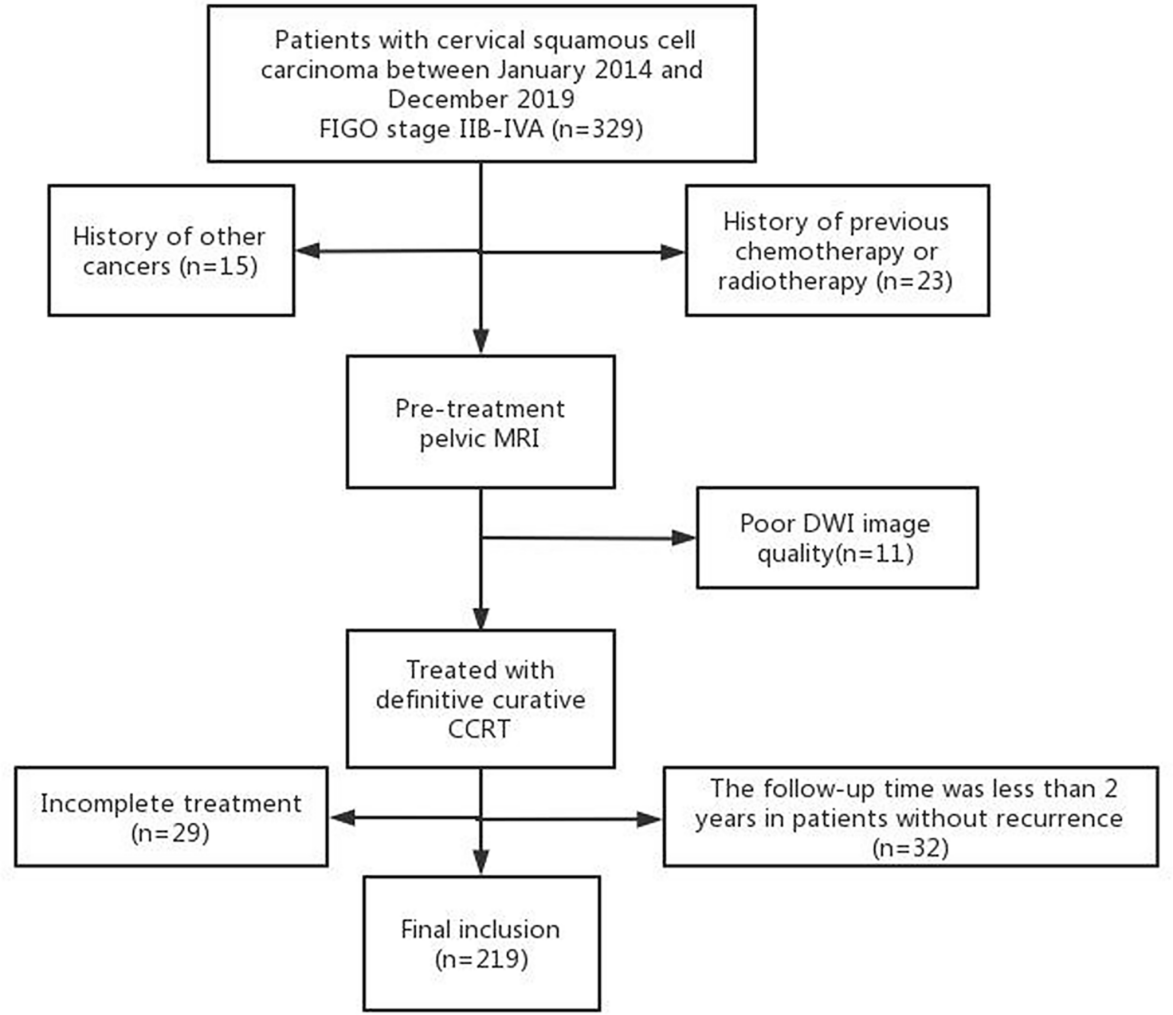
The flowchart of the study cohort.

The institutional review board approved this study and waived the need for informed consent. A retrospective review was performed on patients with cervical cancer treated in our hospital between January 2014 and December 2019. Inclusion criteria were the following: (1) histologically proven cervical squamous cell cancer; (2) FIGO stage IIB to IVA, according to the 2018 FIGO staging system; (3) undergoing pelvic MRI before treatment, including DWI; (4) treated with definitive curative CCRT. Exclusion criteria were: (1) history of other cancers; (2) history of previous chemotherapy or radiotherapy; (3) DWI images could not be evaluated due to artifacts or other factors; (4) failed to complete the treatment; (5) the follow-up time was less than 2 years. The flowchart of the study population is shown in **Figure 1**.

A radiologist with 18 years of experience in gynecological imaging (X. Y.) and a clinician with 20 years of experience in gynecological tumor diagnosis (J. A.) who were blinded to clinical outcomes of the patients restaged all patients based on clinical and imaging examination results using the revised 2018 FIGO staging system. Lymph node with short-axis diameter ≥ 1.0 cm or liquefaction necrosis was assessed as lymph node metastasis (LNM). The two doctors also classified the primary tumor invasion (T stage) in consensus according to TNM 8^th^ edition. The incorporated clinical variables included age, body mass index (BMI), squamous cell carcinoma antigen (SCC-Ag), tumor grade, 2018 FIGO stage, T stage, tumor maximum-diameter, and LNM. Tumor maximum-diameter was determined by the longest diameter measured on T2-weighted imaging (T2WI) in the sagittal or transverse axial planes.

### MR Techniques

All patients underwent routine contrast-enhanced pelvic MRI, including DWI before CCRT. MRI acquisitions were performed on two 3.0-T MR imaging units (Discovery MR 750 and Signa Excite HDx, GE Medical System) using an eight-element phased coil with patients in the supine position. To reduce bowel motion artifacts, patients with no contraindications received an intramuscular injection of 10 mg raceanisodamine hydrochloride before image acquisition. DWI was performed using a single-shot spin echo-planar imaging sequence. The corresponding b values were 0 and 800 s/mm^2^. Sagittal dynamic contrast-enhanced scanning was performed using liver acquisition with volume acceleration-extended volume (LAVA-XV) sequence 15 seconds after an intravenous injection contrast agent (gadodiamide, 0.1 mmol/kg; Omniscan; GE Healthcare, Co. Cork, Ireland) at a rate of 2.0 ml/s, per phase of 15 seconds with a total acquisition time of 105 seconds, followed by 20 mL of normal saline to flush the tubing. Detailed information on the MR sequences is listed in **Table 1**.

**Table 1:** MR imaging parameters.

Sequence	Imaging Plane	TR(ms)/TE(ms)	SliceThickness(mm)	Gap(mm)	Field of View(mm)	Acquisition matrix (phase * frequency)	Number of Excites	b-values (sec/mm^2^)
**GE signa excite HD 3.0T**								
FSE T1WI	Axial	520/7.6	5	1	400	320*224	2	–
FS FSE pelvic T2WI	Axial	5400/106.5	5	1	400	320*256	2	–
FS FSE retro T2WI^a^	Axial	4900/100.2	5	1	400	320*256	2	–
FSE T2WI	Sagittal	4900/157	4	0.4	240	320*256	1	–
FSE T2WI	Axial oblique	5200/134.6	4	1	220	320*256	1	–
Diffusion-weighted	Axial	4700/64.4	5	1	400	128*128	2	0, 800
DCE	Sagittal	3.8/1.8	3	0	300	320*192	0.7	–
**GE Discovery HD750 3.0T**								
LAVA-Flex T1WI	Axial	4.2/1.9	3	0	380	320*224	0.7	
FS FSE pelvic T2WI	Axial	4734/90.3	5	1	380	320*256	2	
FS FSE retro T2WI^a^	Axial	4734/87.9	5	1	380	320*256	2	
FSE T2WI	Sagittal	5907/126.4	4	0.4	240	320*256	1	
FSE T2WI	Axial oblique	6241/121.6	4	0.4	200	320*256	1	
Diffusion-weighted	Axial	4000/56	5	1	380	128*128	6	0, 800
DCE	Sagittal	3.8/1.8	3	0	340	320*192	0.7	

a Retro T2WI sequence was performed from the renal hilum level to the first floor of the pelvic axial T2WI sequence to evaluate the retroperitoneal lymph node status.

FSE, fast spin echo; FS, fat suppression; DCE, dynamic contrast enhanced; TR, repetition time; TE, echo time; T1WI, T1-weighted imaging; T2WI, T2-weighted imaging; LAVA, liver acquisition with volume acceleration.

### Treatment

All patients received whole pelvic external pelvic beam radiation therapy (EBRT) or extended-field RT to the para-aortic area depending on their workup at 1.8-2.4 Gy daily, for 5 days a week, with a total dose of 45-61.6 Gy. Subsequent high-dose-rate brachytherapy (HDR-BT) treatments were performed one week after EBRT, with a total dose range between 21 and 47 Gy at 5.6-8.6 Gy per fraction. In addition, all patients received concurrent chemotherapy with weekly cisplatin or nedaplatin at a dose of 50 mg/m^2^ in case of renal contraindication.

### Tumor Image Segmentation

ADC map was derived from DWI by a workstation (Advantage Workstation 4.6; GE Medical System). ITK-SNAP (v.3.6.0; www.itksnap.org) was used for manual 3D segmentation of MR images. The volume of interest (VOI) of the tumor was segmented by manually drawing ROI by one radiologist (X.Z., with 5 years of experience in gynecological imaging), who was blinded to the results of patients’ outcomes. The ROIs were drawn along the margin of the tumor on each slice of DWI images, carefully avoiding cystic, necrotic, or hemorrhagic tumor regions with reference to T2WI and contrast-enhanced images, and were then finally copied into ADC maps. Eighty patients were randomly selected for tumor segmentation by another radiologist with 8 years of experience in gynecological imaging (Q.Z.) to calculate the interobserver agreement and segmentation performance between two radiologists.

### Texture Feature Extraction

Twenty-two first-order texture features were obtained from the segmentation of the whole tumor on the ADC map by using the PyRadiomics (https://pyradiomics.readthedocs.io): ADC_min_, ADC_5%_, ADC_10%_, ADC_25%_, ADC_50%_, ADC_75%_, ADC_90%_, ADC_95%_, ADC_max_, ADC_mean_, interquartile range (IQR), range, mean absolute deviation (MAD), robust mean absolute deviation (rMAD), root mean squared (RMS), energy, total energy, entropy, skewness, kurtosis, variance, and uniformity.

### Follow-Up

Regular follow-up was conducted every third month until 2 years after diagnosis, twice per year for 3-5 years, and once a year thereafter. Recurrence was confirmed by gynecological examination, tumor marker measurements, and imaging modalities. Recurrences were considered as local (vaginal and/or cervical), regional (pelvic/para-aortic), or distant (upper abdominal and/or extra-abdominal). Recurrence-free survival (RFS) was defined as the time from the first fraction of radiation therapy to the disease recurrence or the last visit in follow-up. Patients with persistent disease were considered to have relapsed on the first day when completing CCRT. The recurrence group and non-recurrence group were divided based on RFS ≤ 2 year and RFS > 2 years.

### Statistical Analysis

The interobserver agreement between two radiologists was calculated by using interclass correlation coefficient (ICC), and the agreement between segmentations was assessed by the Dice coefficient. The Kolmogorov-Smirnov test was used to examine whether the data followed a normal distribution. The Levene test was used to test homogeneity of variance. The Mann-Whitney U test or independent sample test-test was used to compare the differences in the continuous variables between groups. The chi-squared test or Fishers exact test was employed for categorical variables. Receiver operating characteristic (ROC) curves were generated with respective cut-off values determined to accommodate the best diagnostic accuracy according to the Youden index. The correlation between the significantly statistical first-order texture features was calculated according to Spearman or Pearson correlation analysis according to their distribution types. Only variable with higher area under the curve (AUC) was collected in high correlation variables when the correlation was ≥ 0.8, while others were deleted. Logistic regression was used for multivariate analysis. The independent risk factor was screened by backward stepwise regression, by which the prediction model and nomogram were established. Delong test was used for comparing the differences in the values of AUC between different models. The differences in 2-year RFS were evaluated using the Kaplan-Meier method with a log-rank test. A p-value < 0.05 was considered statistically significant. All statistical analyses were performed with SPSS Statistics, version 19.0, and R software, version 3.4.4.

## Results

### Patient Characteristics and Clinical Outcome

A total of 219 patients were included in this study (mean age, 52.6 ± 9.0 years; age range, 24-73 years). Patients were randomly divided into a training cohort (n = 153) and a testing cohort (n = 66) at a ratio of 7:3. There were no differences in the clinical variables between the training and testing cohorts (p > 0.05, **Table 2**). All patients were followed up for more than 2 years except those who died within 2 years. The median follow-up duration was 34.8 months (range, 3.4-81.1 months). The 1-year and 2-year RFS rates were 82.6% and 71.7%, respectively. In the training cohort, there were 43 patients in the recurrence group (17 patients with locoregional recurrence, 18 patients with isolated distant recurrence, and 8 patients with both regional and distant recurrence) and 110 patients in the non-recurrence group. In the testing cohort, there were 19 patients in the recurrence group (6 patients with locoregional recurrence, 9 patients with isolated distant recurrence, and 4 patients with both regional and distant recurrence) and 47 patients in the non-recurrence group.

**Table 2:** Comparison of clinical variables between training and testing cohorts.

Parameters	Training (n=153)	Testing (n=66)	p-value
Age (years)	54 (47, 59)	53 (46, 58)	0.552
BMI (kg/m^2^)	24.49 ± 3.34	24.48 ± 3.55	0.987
SCC-Ag (ng/mL)	5.30 (2.10, 15.35)	5.50 (2.08, 11.88)	0.817
Tumor grade (%)			0.665
Low-grade (well-/moderately differentiated)	102 (66.7%)	42 (63.6%)	
High-grade (poorly differentiated)	51 (33.3%)	24 (36.4%)	
2018 FIGO stage (%)			0.152
II	76 (49.7%)	27 (40.9%)	
III	69 (45.1%)	38 (57.6%)	
IVA	8 (5.2%)	1 (1.5%)	
T stage (%)			0.335
T2	116 (75.8%)	49 (74.2%)	
T3	29 (19.0%)	16 (24.2%)	
T4	8 (5.2%)	1 (1.5%)	
Tumor maximum-diameter (cm)	4.61 ± 1.20	4.54 ± 1.14	0.685
LNM (%)			0.534
Negative	85 (55.6%)	35 (53.0%)	
Pelvic LNM	48 (31.4%)	25 (37.9%)	
Para-aortic LNM	20 (13.1%)	6 (9.1%)	

Continuous variables are presented as mean ± standard deviation or median and interquartile range according to their distribution; categorical variables are presented as n (%).

BMI, Body mass index; SCC-Ag, Serum levels of squamous cell carcinoma antigen; FIGO, Federation of Gynecology and Obstetrics; LNM, Lymph node metastasis.

### Analysis of Clinical Variables for Predicting Early Recurrence

**Figure 2:**
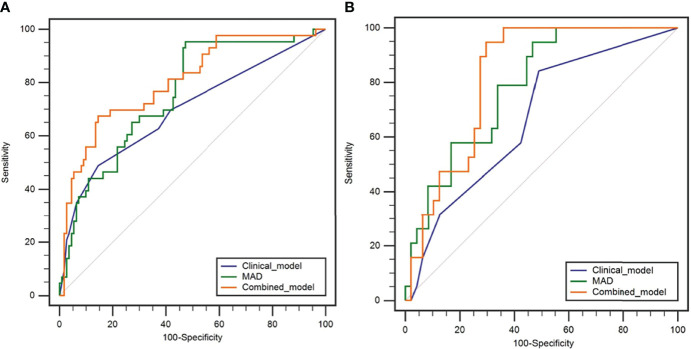
Receiver operating characteristic curves of the clinical model, MAD, and combined model in predicting the early recurrence of LACSC treated with CCRT in the training cohort **(A)** and testing cohort **(B)**.

In the training cohort, there were significant differences in SCC-Ag, FIGO stage, T stage, tumor maximum diameter, and LNM between the recurrence and non-recurrence groups (**Table 3**). There were no significant differences in age, BMI, and tumor grade between the recurrence and non-recurrence groups (all p > 0.05) (**Table 3**). Multivariate logistic analysis showed that T stage (p = 0.071) and LNM (p = 0.007) were independent risk factors (**Table 3**). A clinical prediction model including T stage and LNM was established, and the AUC was 0.697 (95% CI: 0.598-0.797) in the training cohort, with a sensitivity of 48.8% and a specificity of 85.5% (**Table 5**). In the testing cohort, the AUC was 0.667 (95% CI: 0.527-0.806), with a sensitivity of 84.1% and a specificity of 51.1% (**Table 5**). The ROC curves of the clinical prediction model are shown in **Figure 2**.

**Table 3:** Comparison of clinical variables between recurrence and non-recurrence groups in the training cohort.

Parameters	Recurrence (n=43)	Non-recurrence (n=110)	p-value	Multivariate analysis	
				OR (95%CI)	p-value
Age (years)	53 (46, 57)	54 (48, 60)	0.141		
BMI (kg/m^2^)	24.09 ± 2.76	24.65 ± 3.54	0.349		
SCC-Ag (ng/mL)	8.70 (4.70, 21.90)	4.00 (1.80, 10.78)	0.010	–	0.775
Tumor grade (%)			0.162		
Low-grade (well-/moderately differentiated)	25 (58.1%)	77 (70.0%)			
High-grade (poorly differentiated)	18 (41.9%)	33 (30.0%)			
2018 FIGO stage (%)			0.003	–	0.183
II	13 (30.2%)	63 (57.3%)			
III	25 (58.1%)	44 (40.0%)			
IVA	5 (11.6%)	3 (2.7%)			
T stage (%)			0.001	1.91 (0.95, 3.85)	0.071
T2	24 (55.8%)	92 (83.6%)			
T3	14 (32.6%)	15 (13.6%)			
T4	5 (11.6%)	3 (2.7%)			
Tumor maximum diameter (cm)	4.47 ± 1.26	4.96 ± 0.96	0.012	–	0.477
LNM (%)			<0.001	2.19 (1.24, 3.87)	0.007
Negative	16 (37.2%)	69 (62.7%)			
Pelvic LNM	13 (30.2%)	35 (31.8%)			
Para-aortic LNM	14 (32.6%)	6 (5.5%)			

Continuous variables are presented as mean ± standard deviation or median and interquartile range according to their distribution; categorical variables are presented as n (%).

BMI, Body mass index; SCC-Ag, Serum levels of squamous cell carcinoma antigen; FIGO, Federation of Gynecology and Obstetrics; LNM, Lymph node metastasis.

### Analysis of First-Order Texture Features for Predicting Early Recurrence

The ICCs (95% CI) of inter-observer reproducibility for the first-order texture features are listed in the **Supplementary Materials**. The ICCs were all greater than 0.75. The mean Dice coefficient was 0.91 ± 0.05 (range: 0.73-0.97).

In the training cohort, ADC_50%_, ADC_75%_, ADC_90%_, ADC_95%_, ADC_mean_, entropy, IQR, MAD, rMAD, RMS, and variance were significantly lower in the recurrence group compared with the non-recurrence group; kurtosis, skewness, and uniformity were significantly higher in the recurrence group compared with the non-recurrence group (**Table 4**). ADC_90%_, kurtosis, and MAD were incorperated into multivariate logistic analysis after correlation analysis. The result showed that MAD (p < 0.001) was the independent protective factor (**Table 4**), with an AUC of 0.756 (95% CI: 0.673-0.838) in the training cohort and 0.783 (95% CI: 0.671-0.894) in the testing cohort, which were higher than AUCs of the clinical prediction model; however, the differences were not statistically significant (p > 0.05) (**Table 5**). The sensitivity and specificity were 95.3% and 52.7% in the training cohort and 94.7% and 53.2% in the testing cohort, respectively. ROC curves of the MAD are shown in **Figure 2**.

**Table 4:** Comparison of first-order texture features between recurrence and non-recurrence group in the training cohort.

Parameters	Recurrence (n=43)	Non-recurrence (n=110)	p-value	Multivariate analysis	
				OR (95%CI)	p-value
ADC_5%_(10^-3^ mm^2^/s)	0.78 ± 0.09	0.80 ± 0.12	0.292		
ADC_10%_(10^-3^ mm^2^/s)	0.81 ± 0.10	0.83 ± 0.13	0.267		
ADC_25%_(10^-3^ mm^2^/s)	0.87 ± 0.11	0.91 ± 0.14	0.171		
ADC_50%_(10^-3^ mm^2^/s)	0.96 ± 0.13	1.01 ± 0.16	0.047		
ADC_75%_(10^-3^ mm^2^/s)	1.05 (0.95, 1.19)	1.16 (1.06, 1.29)	0.001		
ADC_90%_(10^-3^ mm^2^/s)	1.24 ± 0.18	1.39 ± 0.20	<0.001	–	0.474
ADC_95%_(10^-3^ mm^2^/s)	1.37 ± 0.19	1.53 ± 0.21	<0.001		
ADC_max_(10^-3^ mm^2^/s)	2.05 ± 0.38	2.16 ± 0.36	0.078		
ADC_min_(10^-3^ mm^2^/s)	0.64 (0.56, 0.72)	0.66 (0.58, 0.75)	0.304		
ADC_mean_(10^-3^ mm^2^/s)	1.00 ± 0.13	1.07 ± 0.15	0.007		
Energy(10^9^)	1.92 (1.28, 3.76)	1.62 (0.96, 3.53)	0.230		
Entropy	4.29 ± 0.35	4.56 ± 0.34	<0.001		
IQR	203.00 (164.50, 250.00)	257.50 (203.00, 319.38)	<0.001		
Kurtosis	5.91 (4.69, 7.39)	5.06 (3.85, 6.24)	0.005	–	0.207
MAD	139.92 ± 35.67	180.06 ± 48.42	<0.001	0.98 (0.96, 0.99)	<0.001
Range(10^3^)	1.37 (1.09, 1.64)	1.44 (1.25, 1.79)	0.122		
rMAD	85.80 (71.44, 107.60)	113.62 (89.74, 137.80)	<0.001		
RMS(10^3^)	1.02 ± 0.13	1.10 ± 0.15	0.003		
Skewness	1.45 (1.12, 1.68)	1.35 (1.02, 1.58)	0.092		
Total Energy(10^10^)	2.82 (1.76, 5.06)	2.69 (1.40, 4.87)	0.201		
Uniformity	0.07 ± 0.02	0.06 ± 0.01	<0.001		
Variance(10^4^)	3.42 (2.27, 4.75)	5.22 (3.43, 7.32)	<0.001		

Continuous variables are presented as mean ± standard deviation or median and interquartile range according to their distribution.

ADC, apparent diffusion coefficient; IQR, interquartile range; MAD ,mean absolute deviation; rMAD, robust mean absolute deviation; RMS, root mean squared.

**Table 5:** Performance of models in predicting early recurrence.

Cohort	Model	AUC (95% CI)	Sensitivity%	Specificity%
Training cohort	Clinical model	0.697 (0.598,0.797)	48.8	85.5
	MAD	0.756 (0.673,0.838)	95.3	52.7
	Combined model	0.804 (0.725,0.883)	67.4	85.5
Testing cohort	Clinical model	0.667 (0.527,0.806)	84.1	51.1
	MAD	0.783 (0.671,0.894)	94.7	53.2
	Combined model	0.821 (0.722,0.919)	94.7	70.2

AUC, area under the curve; MAD, mean absolute deviation.

### Establishment of Combined Model for Predicting Early Recurrence

**Figure 3:**
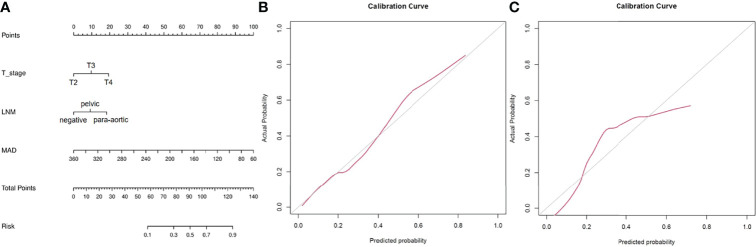
Nomogram and calibration curves for the combined model. **(A)** Nomogram for the combined model. **(B)** The calibration curve of the training cohort. **(C)** The calibration curve of the testing cohort.

**Figure 4:**
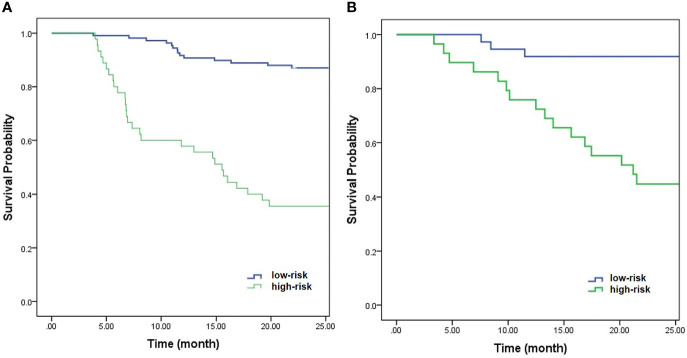
Kaplan-Meier curves of the combined model for 2-year RFS in the training cohort **(A)** (p < 0.001) and testing cohort **(B)** (p < 0.001).

Further multivariable logistic analyses identified the MAD (HR: 0.978; 95% CI: 0.967-0.990, p < 0.001), T stage (HR: 1.902; 95% CI: 0.881-4.107, p = 0.102), and LNM (HR: 1.835; 95% CI: 0.991-3.399, p = 0.054) as independent predictors. The combined model achieved the best predictive performance, with an AUC of 0.804 (95% CI: 0.725-0.883) in the training cohort and 0.821 (95% CI: 0.722-0.919) in the testing cohort, which were significantly higher than those of the clinical prediction model (training: p = 0.003; testing: p = 0.028). The sensitivity and specificity were 67.4% and 85.5% in the training cohort and 94.7% and 70.2% in the testing cohort, respectively. ROC curves of the combined model are shown in **Figure 2**. The nomogram and calibration curves of the combined model are shown in **Figure 3**. The calibration curves of the nomogram showed good agreement between the nomogram-evaluated and actual probabilities of patients with early recurrence. The Hosmer–Lemeshow test yielded nonsignificant p values in the training and testing cohorts (p = 0.42 and 0.18, respectively). The Kaplan-Meier curves for 2-year RFS of the combined model are shown in **Figure 4**.

## Discussion

CCRT is an optimal therapy for LACC with appreciable outcomes, but treatment for relapse of tumor afterward remains tough. One study reported that 62%-89% of cervical cancer recurrences are detected within 2 years of primary treatment (23). It would be of clinical significance to find high-risk patients who are subject to recurrence within a short time, and those patients might benefit from additional or novel therapies, such as targeted agents with chemotherapy (24), or adjuvant or consolidation chemotherapy after CCRT (25). This study demonstrated that T stage, LNM, and MAD were independent predictive factors in predicting recurrence within 2 years in LACSC patients treated with CCRT. The combined model exhibited the highest AUC, which was significantly higher than that of the clinical model, indicating that adding first-order texture features to clinical features could significantly improve the diagnostic performance. Also, all first-order texture features showed good interobserver agreement, which is critical for consideration of model selection and successful clinical implementation.

In this study, only squamous cell carcinoma was included because it is the most common histological type of cervical cancer, and also to exclude possible interference due to different pathology types. Among the included clinical variables, we observed that the SCC-Ag of the recurrence group was significantly higher than the non-recurrence group, which was similar to a previous study (26). The tumor maximum diameter and T stage also showed a distinctive difference between the recurrence and non-recurrence groups in this study. The tumor maximum diameter of the recurrence group was significantly higher than the non-recurrence group, and patients with a higher T stage had a higher risk of recurrence, which was consistent with previous studies (27–29), indicating that local tumor factors remain salient prognostic factors in cervical cancer. Another important factor correlated with recurrence is LNM. The patients with LNM had a higher risk of recurrence in this study, especially those with para-aortic LNM, which was close to previous results that LNM was associated with the recurrence of cervical cancer (21). LNM is one of the most important prognostic indicators for recurrence and death in patients with cervical cancer, and the presence of LNM correlates to a 30%-50% reduction in 5-year survival outcomes (30, 31). Thus, the 2018 FIGO staging system highlights the importance of nodal involvement in cervical cancer patients. Multivariate logistic analysis showed that T stage and LNM were independent risk factors in this study. The AUC of the clinical prediction model was 0.697 in the training cohort and 0.667 in the testing cohort. Accurate estimation of recurrence for patients with LACSC based on clinical variables offers the possibility of tailoring the aggressiveness of treatment to the individual situation, however, the AUC of the clinical prediction model was relatively low. Besides, the model still lacks quantitative parameters reflecting tissue microstructure, which is associated with tumor progression and might be helpful in a more precise prediction of tumor recurrence.

First-order texture features can be obtained from the histogram of pixel intensity values (32). It has been previously reported that pretreatment ADC histogram analysis may serve as a biomarker for predicting tumor recurrence in patients with cervical cancer treated with either surgery or definitive CCRT (11–15). The ADC_50%_, ADC_75%_, ADC_90%_, ADC_95%_, and ADC_mean_ were significantly lower in the recurrence group compared with the non-recurrence group in this study, which was similar to previous studies (12–14). In addition to ADC values, there are other first-order texture features that can describe the distribution of values of individual voxels. Multivariate logistic analysis showed that MAD was an independent protective factor. The MAD was significantly lower in patients with tumor recurrence compared with patients without recurrence.

MAD is the mean distance of all intensity values from the mean value of the image array (33), which represents the uniformity of ADC values in VOI in the present study, with a large MAD indicating a more dispersed ADC distribution in VOI (34). We speculated that the lower MAD in this study might represent areas with a higher proportion of solid tumors and higher cell density and that might be associated with a hostile microenvironment in tumors. Tord et al. (35) found that higher cell density may lead to increased hypoxia and interstitial hypertension, which in turn increased microenvironment-associated metastasis. Moreover, Liu et al. (34) found that poorly differentiated CSCC showed higher MAD. However, they included the necrosis areas of the tumor in delineating the VOI. The result might be related to the significant hypoxia-associated necrosis. The patients included in this study were all with advanced stage CSCC, and most of the tumors contained necrotic areas. Considering that the ADC map might be confounded by the highly elevated ADC values seen in necrotic areas, the necrotic areas were excluded according to T2WI and contrast-enhanced images in this study to avoid the influence of tumor heterogeneity caused by necrosis. MAD has satisfactory prediction performance in both the training and testing cohorts and is superior to the clinical model, indicating that MAD has the potential to predict the early recurrence of LACSC. This study also constructed the combined model, with the addition of MAD in the clinical model, the predictive performance was significantly improved, which showed the highest AUC in both the training and testing cohort.

This study had several limitations. First, the selection criteria of metastatic lymph nodes might lead to the direct exclusion of patients with smaller metastatic lymph nodes, or the included metastatic lymph node had a false positive. Secondly, this study was a single-center and retrospective study. There was inevitable selection bias and referral bias. Thus, external validation of the prediction model is extremely important to test the prognostic significance. Thirdly, the follow-up was relatively short. A longitudinal study is needed to further evaluate the long-term predictive value of the model. Fourthly, only two b values (b-values of 0 and 800 s/m²) were applicated in the present study. Deviation may exist in the result we gained, therefore warrants continued investigations with different b values.

## Conclusion

This study showed that first-order texture features extracted from the whole-tumor texture analysis of the ADC map had the potential to predict the early recurrence of patients with LACSC treated by CCRT. The combined model including MAD, T stage, and LNM could significantly improve the prediction performance, which might provide a new insight for clinicians in tailoring treatment to more curative and counseling patients regarding prognosis.

## Data Availability Statement

Data used in this study are not publicly available and can only be accessed, with appropriate approvals from data custodians and ethical clearance, from National Cancer Center/National Clinical Research Center for Cancer/Cancer Hospital, Chinese Academy of Medical Sciences and Peking Union Medical College. Requests to access the datasets should be directed to XinZ, xinmingzh@sina.com.

## Ethics Statement

The studies involving human participants were reviewed and approved by National Cancer Center/National Clinical Research Center for Cancer/Cancer Hospital, Chinese Academy of Medical Sciences and Peking Union Medical College. The ethics committee waived the requirement of written informed consent for participation.

## Author Contributions

XiaoZ, XY, and XinZ: conception and design and administrative support. XiaoZ, XY, QZ, and JA: collection and upload of data. XiaoZ, QZ, LX, and SW: data analysis and interpretation. XiaoZ wrote the first draft of the paper; XY and XinZ revised the article. All authors contributed to the article and approved the submitted version.

## Conflict of Interest

Authors LX and SW were employed by GE Healthcare.

All authors declare that the research was conducted in the absence of any commercial or financial relationships that could be construed as a potential conflict of interest.

## Publisher’s Note

All claims expressed in this article are solely those of the authors and do not necessarily represent those of their affiliated organizations, or those of the publisher, the editors and the reviewers. Any product that may be evaluated in this article, or claim that may be made by its manufacturer, is not guaranteed or endorsed by the publisher.
