# Supplementary material for: The Value of Whole-Tumor Texture Analysis of ADC in Predicting the Early Recurrence of Locally Advanced Cervical Squamous Cell Cancer Treated With Concurrent Chemoradiotherapy
Source: Front Oncol. 2022 May 20;12:852308. doi: 10.3389/fonc.2022.852308 (PMC9165468; doi:10.3389/fonc.2022.852308)
Supplement: Supplementary file 1 [file Table_1.docx]

**Table 1** The ICC (95 % CI) of inter-observer reproducibility from two radiologists.

|  | ICC | 95 % CI |
| --- | --- | --- |
| ADC_5%_ | 0.999 | 0.998-0.999 |
| ADC_10%_ | 0.998 | 0.998-0.999 |
| ADC_25%_ | 0.997 | 0.996-0.998 |
| ADC_50%_ | 0.988 | 0.981-0.992 |
| ADC_75%_ | 0.964 | 0.944-0.977 |
| ADC_90%_ | 0.919 | 0.874-0.948 |
| ADC_95%_ | 0.908 | 0.857-0.941 |
| ADC_max_ | 0.755 | 0.617-0.843 |
| ADC_min_ | 0.984 | 0.975-0.990 |
| Mean | 0.977 | 0.964-0.985 |
| Energy | 0.984 | 0.975-0.990 |
| Entropy | 0.936 | 0.901-0.959 |
| IQR | 0.929 | 0.889-0.954 |
| Kurtosis | 0.752 | 0.613-0.841 |
| MAD | 0.899 | 0.843-0.936 |
| Range | 0.795 | 0.681-0.869 |
| rMAD | 0.916 | 0.869-0.946 |
| RMS | 0.969 | 0.952-0.980 |
| Skewness | 0.884 | 0.819-0.926 |
| Total Energy | 0.984 | 0.975-0.990 |
| Uniformity | 0.959 | 0.936-0.974 |
| Variance | 0.885 | 0.821-0.926 |

*ICC* interclass correlation coefficient, *ADC* apparent diffusion coefficient, *IQR* interquartile range, *MAD* mean absolute deviation, *rMAD* robust mean absolute deviation, *RMS* root mean squared
